# Highly cadmium tolerant fungi: their tolerance and removal potential

**DOI:** 10.1186/s40201-015-0176-0

**Published:** 2015-03-14

**Authors:** Mehran Mohammadian Fazli, Negin Soleimani, Mohammadreza Mehrasbi, Sima Darabian, Jamshid Mohammadi, Ali Ramazani

**Affiliations:** Department of Environmental Health Engineering, Zanjan Universiry of Medical Sciences, Zanjan, Iran; Medical Entomology and Mycology Department, School of Medicine, Zanjan Universiry of Medical Sciences, Zanjan, Iran; Biotechnology Departments, School of Pharmacy, Zanjan University of Medical Sciences, Zanjan, Iran

**Keywords:** Cadmium tolerance, Bioremediation, Fungi

## Abstract

**Background:**

Soil and effluent of lead and zinc industries contain high concentration of cadmium. The present study was conducted to isolate tolerant fungal strains from cadmium -polluted sites in Zanjan province, Iran.

**Methods:**

Cadmium tolerance and bioremediation capacity of seven isolates including *Aspergilus versicolor, Aspergillus fumigatus, Paecilomyces* sp.9*, Paecilomyces* sp.G*, Terichoderma* sp*, Microsporum* sp*,Cladosporium* sp were determined.

**Results:**

Minimum inhibitory concentration values among 1,000-4,000 mg lˉ^1^proved great ability of isolated strains to survive in cadmium polluted environments*.* The most tolerant fungi, *Aspergilus versicolor*, showed tolerance index of 0.8 in 100 mg lˉ^1^ cadmium agar media. Fungal resistance against cadmium is depended directly on strain’s biological function.

*A. versicolor* was found to bioaccumulate over7 mg of cadmium per 1 g of mycelium, followed by 5.878, 5.243, and 5.075, 4.557 by *Paecilomyces* sp*, Aspergilus fumigatus, Microsporum* sp *and Terichoderma* sp, respectively.

**Conclusion:**

It can be noted that tolerance of the strains appears to be independent from bioaccumulation capacity. Finally, the results indicated that *A. versicolor* could be a prospective candidate for bioremediation processes.

## Background

Human jump toward industrialization and comfort life is a leap into environmental pollution and consequently deterioration of human health. The environment is polluted by heavy metals from industrial wastewaters during metal processing as well as other pollutant routes. Virtually, any industrial activity using metals has a metal disposal problem [1]. Nature of heavy metals is non-biodegradable and persistent; therefore, environmental compartments (soil and water body) are not able to purify themselves from these toxic pollutants.

Heavy metals can be divided into essential metals such as copper, manganese, zinc, and iron, and nonessential metals such as cadmium, lead, mercury, and nickel [2]. Cadmium and lead are included among the major pollutants because of their high toxicity [3-6]. Cadmium is released to ecosystem by mine tailing, effluents from textile, leather, tannery, electroplating and galvanizing industries, as well as cadmium batteries.

Biomagnification of cadmium in nature and migration through drinking water, food and air to human body cause severe health effects like kidney damage, bronchitis and cancer [3]. As industrial development train is not stoppable, struggle with heavy metal pollution requires novel remediation methods. Conventional treatment systems have failures which include insufficient metal sequestration, high costs, high reagents and/or energy requirements, and generation of toxic sludge or other waste products that require disposal. Restoring metals in an efficient and economical procedure has necessitated the use of different options in metal-separating methods. Research shows that bioaccumulation of metals by organisms has been successful to some extent [3]. Bioremediation of heavy metals from aqueous solutions is a relatively new process that has been confirmed as a promising process in the removal of heavy metal pollution. The major advantages of biosorption are its high effectiveness in reducing the heavy metal ions, and the use of inexpensive biosorbents. Biosorption processes are particularly suitable to treat dilute heavy metal wastewater [7].

Biomass obtained from different sources is used in biotreatment and its key feature and potential controlled for the process. Rather than searching thousands of microbial species for particular metal sequestering features, it is beneficial to look for biomasses that are readily available in large quantities to support potential demand. While choosing of the biomaterial for metal sorption, its origin is a major factor to be taken into account [8].

Among biological sources, fungi possess a number of advantages including producing great biomass, rapid growth, availability, and flexibility to rough circumstances.

The uptake of metals by fungal biomass appears to involve a combination of two processes: bioaccumulation (i.e. active metabolism-dependent processes, which may include both transport into the cell and partitioning into intracellular components) and biosorption (i.e. the binding of metals to the biomass by processes that do not require metabolic energy) [9].

Fungi are ubiquitous members of subaerial and subsoil environments, and often become a dominant grouping metal-rich or metal-polluted habitats [10]. Recent studies have shown that the strains isolated from contaminated areas have remarkable potential to tolerate such toxic conditions. Microorganisms have been shown to possess ability to survive by adapting or mutating at high concentrations of heavy metals [11,12].

This could play a key role to explore newcomer resistant isolates from pool of biomasses. Exhibiting high tolerances to heavy metals, these isolates were selected for bioremediation studies. Generally, fungi have often been proposed as bioagents for metal recovery processes [13].

This study was carried out to isolate fungi from cadmium- contaminated sites from Zanjan province of Iran for the first time and evaluate their resistance level toward cadmium as well as assessing their bioaccumulation capacity in order to expand knowledge about bioremediation science.

## Materials and methods

### Materials

The aqueous solutions of cadmium were prepared by diluting Cd (II) standard stock solution (concentration 1000 mg L^-1^) obtained by dissolving Cd (NO_3_)_2_.4H_2_O in deionized water . Fresh dilutions were prepared for each experiment. Cd (NO_3_)_2_.4H_2_O was purchased from Merck, Germany. Potato dextrose agar (PDA), malt extract agar (MEA) and potato dextrose broth (PDB) were used as solid and liquid medium, respectively. PDA and MEA medium were purchased from Merck, Germany. PDB medium was purchased from Scharlau, European Union. Deionized water was used in all experiments (TKA Smsrt2Pure, Germany). All laboratory glassware and plastic were soaked in 2 M HNO_3_ technical grade, rinsed with distilled water and heat dried 2 h, at 180°C before use.

### Sampling and experimental sites

The research areas characterized in this study were lead and zinc refinery industries as contaminated sites and municipal wastewater treatment plant as control site located in Zanjan province, Iran. These locations were selected based on their cadmium pollution densities. Soil samples were taken from six different spots to a depth of 20 cm from waste dumping areas. Effluent samples of industries were collected from wastewater discharge of plants. Control sample was obtained from aeration tank of municipal wastewater treatment plant. Samples were placed in sterilized glass bottles, transported on ice (4°C), taken to the laboratory and analyzed within 8 h. The wastewater and soil samples were analyzed for total content of Cd. The soil samples were dried at 105°C manually ground and sieved (500 μm pore size). One g of soil samples were digested with 70% HNO_3_ (1 M) and 30% H_2_O_2_ using microwave digestion (Sineo MDS-10, China). Cadmium concentrations were analyzed by a Varian Atomic Absorption Spectrophotometer (AA 240, Australia) [14].

### Isolation of strains

Fungal strains were isolated on PDA and MEA by serial dilution method in order to avoid overlapping colonies. Streptomycin (15 mg lˉ^1^) and chloramphenicol (50 mg lˉ^1^) were added to mediums after autoclaving at 15 psi for 15 min and 121°C to arrest bacterial growth. The soil samples (1 g) were suspended in 100 ml of sterilized water. The mixture was shaken (200 rpm) for 30 min at room temperature. All samples were diluted up to (10ˉ^3^). 0.1 ml of different dilutions were spread on Petri plates (diameter 10 cm) containing 20 ml media. Plates were incubated at 28°C in dark condition and monitored every day up to 10 days and each developed colonies were sub-cultured and isolated into fresh PDA plates. Purified isolates were kept on slants at 4°C and recultured every 4 weeks [15].

### Screening for Cd- tolerant fungi

In order to select Cd- tolerant strains, 100 mg lˉ^1^Cd stress was added to PDA medium. The pH of the solid growth medium was adjusted to 6 with 1 M sodium hydroxide solutions before autoclaving in all experiments in this study. The small agar plugs with young mycelium from the edge of the stock cultures were cut and transferred to surface of solid medium. Plates were incubated at 28°C for at least 10 days and visually inspected for microbial growth every day. Cadmium- tolerant strains were subjected to resistance studies.

### Cadmium tolerance index of fungi

Five mm disks from 10 day old pure cultures of each fungal isolates were inoculated into PDA (three replicates) supplemented with 200 mg lˉ^1^ Cd [4]. The inoculated plates were incubated at 28°C for 10 days. In parallel, cultures without cadmium were performed as a control. The radial growth was evaluated from four measurements (in millimeters) that passed through the center of the inoculated portion. The initial diameter of the portion was subtracted from the growth diameter [5]. The mean of perpendicular diameter measurements was recorded for each plate on the day10th. The tolerance index (TI), an indication of the organism response to metal stress was calculated from the growth of strain exposed to the metals divided by the growth in the control plate [16]. The higher the TI, the greater the resistance.

### Determination of minimum inhibitory concentration

Minimum inhibitory concentration (MIC) was defined as the minimum inhibitory concentration of the heavy metal that inhibited visible growth of test fungi [17]. PDA medium was enriched with increasing concentration of Cd (200, 400, 600, 800, 1,000, 2,000, 3,000, and 4,000 mg lˉ^1^Cd). Plates were inoculated with agar plugs from the edge of the 10 day old growing cultures. If no apparent growth of fungi was observed after ten days on the plates, the metal concentration was considered as the highest metal concentration tolerated by the tested fungus.

### Identification of selected fungus

All the resistant fungal isolates were initially identified by colony characteristics on PDA characterized to the genus level on the basis of macroscopic characteristics (colonial morphology, color and appearance of colony, and shape), microscopic characteristics (septation of mycelium, shape, diameter and texture of conidia) and the help of the Principles and practice of clinical parasitology [18]. Molecular identification of cultures was carried out with some modifications by extracting chromosomal DNA of potential fungus according to previous study [19]. Briefly, a pure culture of the isolated fungal strain was grown in liquid shaking PDB medium at 28°C and 120 rpm for 72 h. The biomass was then harvested and washed with sterile distilled water. Cells were broken up by liquid nitrogen, re-suspended in Hoffman Winston extraction buffer; then, proteins were removed with phenol–chloroform and DNA was precipitated by adding pure ethanol. DNA concentrations and *A*260/*A*280 ratio were determined with biophotometer (Biophotometer plus, Eppendorf Germany). An *A*260/*A*280 ratio of 1.8–2.1 was considered acceptable for PCR (polymerase chain reaction)-based procedures. Part of the 18 s rDNA fragments was amplified using primers 817 F and 1536R [20]. The PCR mixture consisted of 25 μl of 2x PCR Master Mix (Fermentase, USA), 10 pM of each primer, 21 μl water and approximately 2 μg genomic DNA as template in a total volume of 50 μL. The PCR was performed in a Thermo cycler (icycler, USA) using a thermal cyclic condition at 94°C (7 min) followed by 8 cycles at 94°C (1 min), 59.5°C (45 s) and 72°C (1:30 min), then 30 cycles with 94°C (1 min), 56°C (45 s), 72°C (1:30 min) with a final extension at 72°C for 10 min. A sample (5 μL) of the PCR product was analyzed by electrophoresis in 2% agarose gel with 1xTBE buffer. Electrophoresis was performed at 80 V for 40 min. The purified amplicons were sequenced by using an automated sequencer (Bioneer, Korea). The sequences were compared using the BLAST program (http://www.ncbi.nlm.nih.gov/BLAST/) for identification of the isolates [21].

### Cadmium bioaccumulation by active fungus

To determine the bioaccumulation ability of the 7 fungal isolates, inoculums (six 5 mm disks of mycelia strain) were prepared from 10-day- old pure fungal culture and inoculated into 250 ml Erlenmeyer flask containing 100 ml potato dextrose broth (PDB) plus 100 mg lˉ^1^ cadmium. Initial concentrations of Cd (II) in each conical flask were checked by AAS before fungal inoculation. pH was adjusted to 6 [22]. Un-inoculated controls (PDB medium with 100 mg lˉ^1^ of Cd and without any fungal inoculums) were served to detect any possible abiotic Cd (II) reduction brought about by media components. All flasks were incubated at 28°C on a rotary shaker at 120 rpm in dark conditions.

After 10 days of incubation (logphase ([23], flasks containing fungal biomass were harvested and filtered through Whatman No.42 filter paper. Filtered PDB medium was used for determining total Cd concentration.

Biomass samples were rinsed three times with distilled water and dried in hot air oven at 80°C until a constant weight (24 h) was achieved. The dried fungal biomass was weighed and defined as dry biomass (g) [24]. The amount of heavy metal uptake (*q*, mg/g) was calculated by using the following equation [25]:

1 $$ q=\left[{C}_i-{C}_f/m\right]V $$

In above equation, *q* (mg/g) is mg of metal ions uptake per gram biomass; *C*i (mgLˉ^1^) is the initial metal concentration of liquid phase; *C*_f_ (mgLˉ^1^) is the final metal concentration; *m* (g) is the amount of dry biomass; and *V* (L) is the volume of the medium.

### Data analysis

All experiments were carried out by triplicate sample. Values reported in this paper are the means ± S.D. The difference in TI and uptake capacity of each isolate was studied by one-way ANOVA followed by post-Hoc multiple comparisons by Duncan’s method using SPSS16 (USA, I1, Chicago, SPSS Inc.). The difference was considered as significant when *P* < 0.05.

## Results and discussion

### Sites characteristic

The range of Cd in earth soils lie between 0.2 and1.1 mg kgˉ^1^. The highest Cd concentrations (in mg kgˉ^1^) are reported for soils in the vicinity of metal-processing industries, for example, in Belgium, 1781; in Poland, 270; and in the United States, 1500 [26].

Table 1 demonstrates Cd content of environmental samples. Cd concentrations of soil samples used in this study were 56.90and 488.25 (mg kgˉ^1^soil) for lead refinery industry and zinc refinery industry respectively. Since dumping waste areas were selected for soil samples, it is not surprising that Cd content was too high.

**Table 1:** **Cadmium concentration of samples and number of resistant isolates**

**Sites**		**Cadmium concentrationª**	**Number of resistant isolates**
**Soil(mg kgˉ** ^**1**^ **of soil)**	Industrial dumping area (lead refinery)	56.9 ± 0.55^a^	5
	Industrial dumping area (zinc refinery)	488.25 ± 1.92	3
**Wastewater(mg l ˉ** ^**1**^ **)**	Industrial effluent(lead refinery)	50.23 ± 1.05	3
	Industrial effluent(zinc refinery)	70.65 ± 1.67	4
	Municipal wastewater treatment plant (aeration tank)	0.23 ± 0.09	1

ªMean ± SD.

The Cd concentration in effluent of mentioned industries were 50.23and 70.65 (mg l ˉ^1^) respectively, whereas Cd in aeration tank of municipal wastewater treatment plant was 0.23 (mg l ˉ^1^). Clearly these values are 500 times higher than Industry Effluent Guidelines regulated by EPA [27]. These results could be due to the fact that Zanjan province is located close to two major heavy metal processing plants in Iran, as well as neighboring to Mahneshan heavy metal mine.

### Number of cadmium- resistant isolates and their origin

As shown in Table 1, sixteen strains could tolerate 100 mg l ˉ^1^ Cd toxicity. These strains were from different sampling sites.

It is known that microorganisms isolated from natural environments contaminated with heavy metals often exhibit tolerance to heavy metal pollutants [28].

Cadmium stress exerted in this study to isolated fungus from municipal wastewater treatment site, made non-favorable lethal growth medium. Discrepancy in conditions the fungi were adapted to resulted in extinction of isolated fungal population from municipal wastewater. Only one resistant isolate was obtained from low- polluted area. In contrast, the number of resistant isolates from heavy metal industrial sites was significant. It is well known that a long-time exposure of water and sediment to heavy metals can produce considerable modification of their microbial populations, reducing their activity and their number. Generally, pollution of soil and water by heavy metals may lead to a decrease in microbial diversity. This is due to the extinction of species sensitive to the stress imposed, and enhanced growth of other resistant species [29].

### Cadmium-resistant assays minimum inhibitory concentration

MIC border line of 1000 (mg l ˉ^1^) was chosen. Strains with MIC value over 1000 (mg l ˉ^1^) were subjected to molecular identification. Approximately 700 bp PCR product of 18S rDNA gene was collected by PCR amplification program. The 18S rDNA gene of seven strain deposited in the GenBank database of NCBI under accession numbers KM205077, KM205078*,* KM205079, and KM205080*.* Data of top seven MIC values and their relevant isolate identification are shown in Table 2. Results from Table 2 show that soil natural sources trigger microorganisms with higher tolerance ability than aquatic sources. Except *Terichoderma* sp, there was no strain from industrial effluent which could survive in over 1000 (mg l ˉ^1^) Cd. Evolutionary adaptation to metal-contaminated soils is a well-documented phenomenon, particularly because it is one of the most striking examples of microevolution driven by edaphic factors [30].

**Table 2:** **Fungi isolated from different sites and their MIC values**

**Origin**	**strain**	**MIC (mg l ˉ** ^**1**^ **)**
Industrial dumping area (lead refinery)	*Paecilomyces* sp.9	4000
	*Paecilomyces* sp.G	4000
	*Microsporum* sp	1000
	*Aspergillus fumigatus*	1000
Industrial dumping area (zinc refinery)	*Cladosporium* sp	1000
	*Aspergilus versicolor*	2000
municipal waste water treatment plant (aeration tank)	*Terichoderma* sp	2000

Cadmium concentration in sites and the levels of survival against Cd toxicity were not related to each other, as *Terichoderma* sp was able to survive up to 2000 (mg l ˉ^1^) Cd toxicity which is 10000 orders of magnitude than Cd concentration in urban wastewater. Other examples are about *Paecilomyces sp.9* and *Paecilomyces* sp.G from lead refinery industry which were able to grow up to 4000 (mg l ˉ^1^) Cd stress, whereas strains from zinc refinery industry with Cd concentration over eight order of magnitude in soil than lead refinery industry only could tolerate up to 2000 and 1000 (mg l ˉ^1^) Cd. It implies that some microorganisms without any consideration about their native root could develop ability to keep up living in severe toxic environments. The MIC values suggested that the resistance level against individual metals was dependent on the isolates [31].

The cadmium concentration of 4000 (mg l ˉ^1^) was the highest MIC value in this study that *Paecilomyces* sp.G and *Paecilomyces sp.9* could tolerate. To a lesser degree were *Aspergillus versicolor* and *Terichoderma* sp with MIC value of 2000 (mg l ˉ^1^) and *Microsporum* sp, *Cladosporium* sp*, Aspergillus fumigates* with MIC of 1000 (mg l ˉ^1^).

There are some studies supporting the idea that there is a very little difference in metal tolerance between strains from polluted and unpolluted sites [32]. Indeed, presence of metal may act as a fatal toxicity on microorganism population, but does not have any influence on microbial tolerance ability.

MIC values of 0.328 mM for filamentous fungi and 1 (mg l ˉ^1^) for *Aspergillus*, *Penicillium* and *Fusarium* were reported [29,33]. In another study no determinations were made for cadmium since the majority of the tested fungi were unable to grow in the presence of this metal [34]. In a study conducted in 2007, MIC values of 5,000, 3,000 and 4,000 (mg l ˉ^1^) Cd were reported for Rhizopus sp., *Terichoderma* and *Aspergillus,respectively*. These reported MIC values are relatively similar to the values we observed [31].

Considering previous studies, *Paecilomyces* sp.G *and Paecilomyces* sp.9 are newly introduced fungus with remarkable tolerance potential.

### Tolerance index

**Figure 1:**
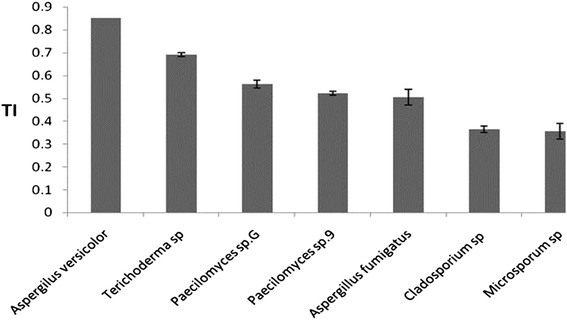
**TI of fungus under 200 mg lˉ** ^**1**^ **of cadmium**. Cadmium tolerance index of different isolates is significantly different (*P* < 0.05). The line on each bar is the standard deviation.

The reduced tolerance index reflects the inhibitory growth function of heavy metal [35]. To select the most tolerant fungi, the actual resistant potential of fungi must be tested. Tolerance level of fungi can be revealed through both TI and MIC assays. Although it is crucial for scientists to discover a fungus with great ability to survive in extremely high heavy metal concentration, from an environmental engineer view, that fungus is salient and applicable for purification systems which in response to metal toxicity could grow and multiply faster. It implies that the more rapidly the fungi can adapt to polluted environment and develop its colonies, the more beneficial it is for treatment process. The term adaptation speed is an important armor that prompts one fungus more powerful than other fungi with higher MIC property. TI of each fungus in this study (Figure 1) demonstrated different orders of tolerance, as follows: *Aspergilus versicolor* and *Terichoderma* sp have shifted up to first level of tolerance with TI of 0.85 and 0.69 respectively, followed by *Paecilomyces* sp.G*, Paecilomyces sp.9* and *Aspergillus fumigatus* with TI of 0.56, 0.52 and 0.5 respectively which were fairly tolerant fungi and finally at end of the list were *Cladosporium* sp (0.36) and *Microsporum* sp (0.35).

*Aspergilus versicolor* from highly polluted zinc industrial site posed minimal reduction in growth (%15). It might be due to the high concentration of cadmium in soil of strain’s original environment that induced resistance strategy on fungal metabolism; therefore *Aspergilus versicolor* was able to adjust rapidly to polluted culture media. Based on the result, *Aspergilus versicolor* was a highly adaptable fungus in response to cadmium stress. Excessive Cd in soil could trigger the evolution for higher Cd tolerance in *Suillus luteus* [36]. An exceptional fungus was *Terichoderma* sp again. Despite the fact that this fungus was from least polluted area with % 31 reductions in growth, it adapted better than five remaining fungi. Trying to find rigid general regulation between the microorganism’s origin and fungal resistance to heavy metal is cumbersome effort. The trustworthy theory is: fungal resistance to heavy metal is depended directly on biological function of the strain. The Cd-resistance was found to be independent from the pollution level at the site of origin [29,32,37]. TI results in this study illustrated that *Aspergilus versicolor* was the most tolerant and *Microsporum* sp and *Cladosporium* sp with 65% suppression of mycelia growth were the most sensitive fungus.

Results from relative study showed that Cadmium at concentration of 1 mM posed the strongest inhibition toward isolates from the genera *Aspergillus*, *Fusarium*, *Alternaria* and *Geotrichum*. Only *Penicillium* isolates expressed tolerance index of 0.8 [34]. In another study, growth of *Aspergillus flavus* was inhibited by 40% at 1 mM Cd concentration [38].

Copious heterogeneity in TI of the isolates, especially in strains of the same genus (*Aspergilus versicolor*, *Aspergillus fumigatus*) and (*Paecilomyces* sp.9*, Paecilomyces* sp.G) proved the theory that various genera and also isolates of the same genus do not necessarily have the same heavy metal tolerance [25,31,34,39]. It comes into mind that tolerance skill is not inherited among microorganisms, in other word it is acquired from ecosystem.

### 3.4. Various tolerance strategies related to different morphological alteration

On exposure to cadmium, morphological changes were observed in all isolated fungi. Several authors have reported the formation of colorful mycelia in the presence of heavy metals on agar media [28].

**Figure 2:**
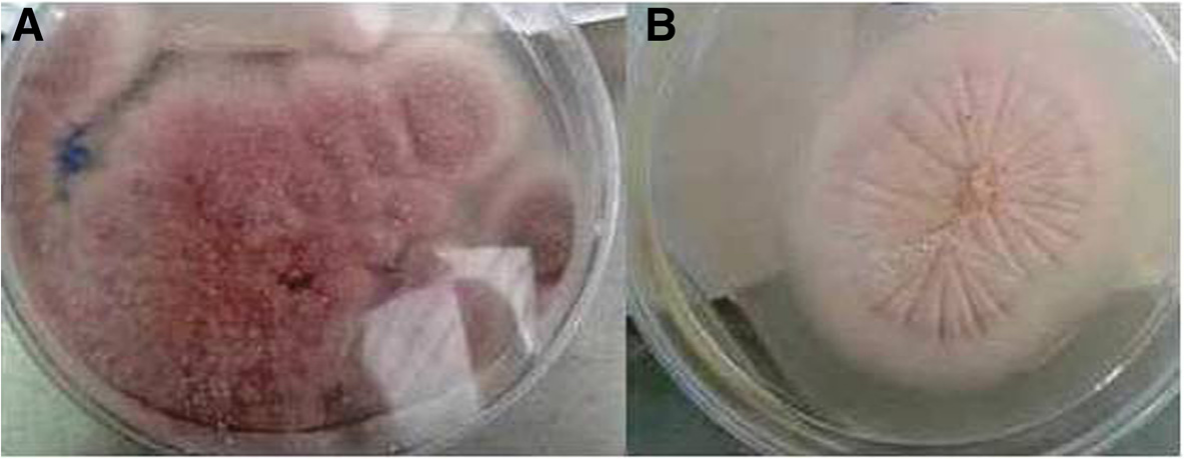
***Paecilomyces*** **sp.G in control plate (A), and in presence of 200 mg lˉ** ^**1**^ **Cd after 7 days (B).**

**Figure 3:**
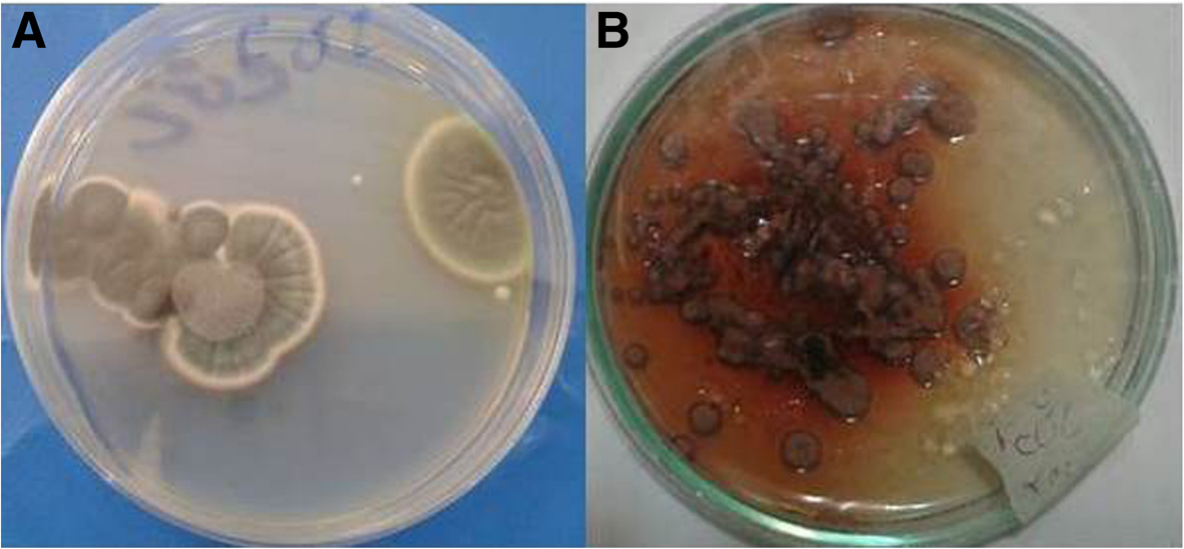
***Aspergilus versicolor*** **in control plate (A), and in presence of 200 mg lˉ** ^**1**^ **Cd after 7 days (B).**

Other than *Aspergilus versicolor* and *Cladosporium* sp genera, decoloration of fungus occurred by increasing the cadmium concentration in medial growth. Pink color changed to white in *Paecilomyces* sp. and *Paecilomyces* sp.9 genera (Figure 2). In *Terichoderma* sp and *Aspergillus fumigatus*, green color changed to white. Red color appeared in cracked mycelium of *Aspergilus versicolor* and sides of its colonies in media were polluted with Cd (Figure 3). Red pigmentation in *Aspergilus versicolor* was probably due to the binding of Cd to the protein in the cell wall of mycelium. It has been previously suggested that production of pigments in fungal cell and cell free media is accompanied with precipitation of metal ions on the call wall [23]. Consistent with this study, *Sills hirsutum* produced a yellow-orange pigment both extracellular and in the mycelium, when cultivated in the presence of 0.25 mM or more Cd [40]. Jarosz-Wilkołazka et al. reported that the presence of Cd induced formation of orange–brown pigment which colored the fragile mycelium of *Abortiporus biennis*, as well as the cell-free culture medium [41].

**Figure 4:**
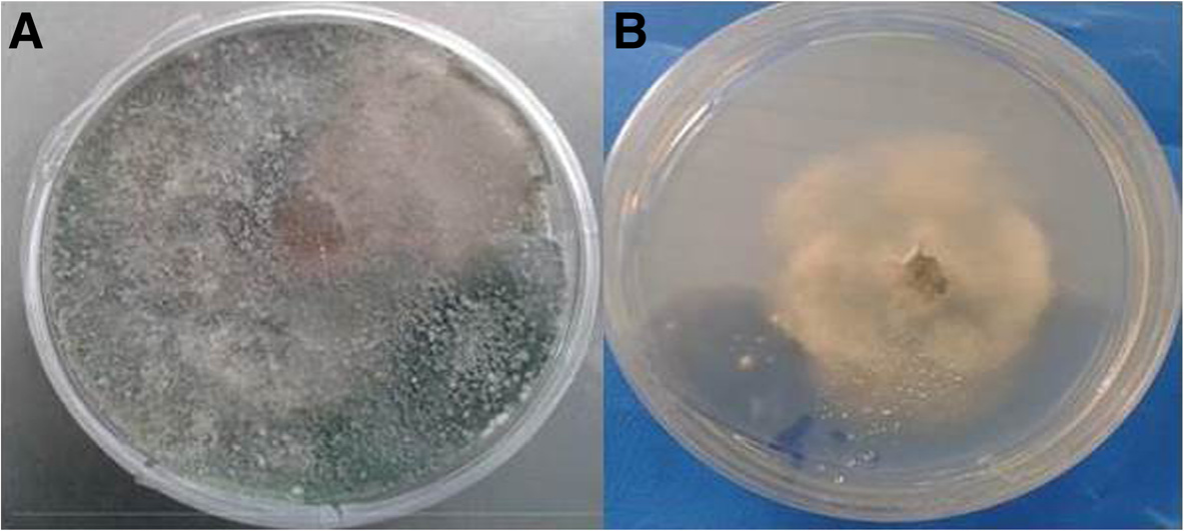
***Terichoderma*** **sp in control plate (A), and in presence of 200 mg lˉ** ^**1**^ **Cd after 7 days (B).**

The appearance of condensed, frizzy mycelium was clearly visible in *Aspergillus fumigatus, Cladosporium* sp*, Microsporom* sp, *Paecilomyces* sp.G*, and Paecilomyces* sp.9 generas. In the case of a toxic metal-containing domain, aggregated mycelia could produce high local concentrations of extracellular products such as complexing agents, precipitating agents, polysaccharides and pigments with metal-binding abilities [42]. In *Terichoderma* sp, isolated in this study, aerial mycelium vanished (Figure 4). On Cd-containing agar most of the mycelium of *Paxillus involutes* grew submerged rather than on the surface as occurs on Cd-free agar [40].

Possible explanation for these different morphological changes among isolates may be due to the vast detoxification/tolerance mechanisms that each strain applies. The variation in the metal tolerance might be due to the presence of one or more types of tolerance strategies or resistance mechanisms exhibited by different fungi [31].

### Bioremoval of cadmium by fungal isolates

**Figure 5:**
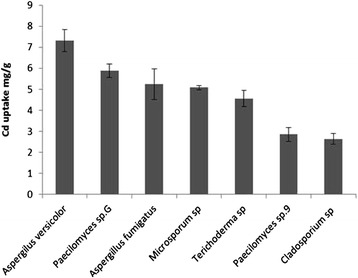
**Metal bioaccumulation of the fungi under 100 mg lˉ** ^**1**^ **of Cadmium, the uptake of cadmium by different isolates is significantly different**. (*p* < 0.05). Bars represent the standard deviation (n = 3).

There has been steady progress in studying the biosorption of heavy metals, resulting in the identification of some biomass types that show very promising uptake of metallic ions [43]. The biosorbents used in heavy metal biosorption are usually obtained after screening the heavy metal resistant/tolerant microorganisms from polluted environments [14]. Adaptation of fungal isolates to heavy metal successfully created organisms with greater efficiency in bioaccumulation [35]. Because the putative specific resistance mechanism(s) could have a potential for biomitigation of contaminated sites, the metal sequestration capacity of the fungus was evaluated [44]. Cadmium bioaccumulation (mg of cadmium uptake per g of dry biomass) of all the tested fungal isolates from liquid media containing 100 mg lˉ^1^ of Cd is presented in Figure 5.

The genera, *Aspergilus versicolor, Paecilomyces* sp.G*, Aspergillus fumigatus, Microsporum* sp*, Terichoderma* sp*, Paecilomyces* sp.9 *and Cladosporium* sp *showed* the bioaccumulation capacities of 7.313, 5.878, 5.243, 5.075, 4.557, 2.849 and 2.631 mg gˉ^1^ in sequence of decreasing the potential. Surprisingly the best accumulator fungus was the most tolerant strain too. However, for the rest of the fungus this trend was not continued. Except *Paecilomyces* sp.G that appeared semi accumulator and semi tolerant, *Aspergillus fumigatus* and *Microsporum* sp were among the sensitive isolates; however, they were moderately accumulator fungi. These results suggest that removal capacity is not proportional to level of tolerance. Similar observations regarding the lack of correlation between metal tolerance and removal capacity have been reported earlier [25,31,32]. Indeed; uptake capacity was related to the type of tolerance mechanism of fungi. In biotreatment criteria, the resistant mechanism and remediation strategies of microorganism should be distinguished and the parts that these topics have in common be selected. Going through mechanisms of tolerance that finally leads to discovering new biouptake activities (bioaccumulation and biosorption) is essential in this field.

A diversity of specific metal accumulation strategies has been known. The physicochemical properties of metals and the physiology of the organism both influence metal uptake [45]. It can be hypothesized that diminished uptake in *Cladosporium* and *Paecilomyces* sp.9 generas contributed to cadmium rejection mechanism of tolerance utilized by this fungus. Fungi are able to restrict entry of toxic metal species into cells by reduced metal uptake and/or increased metal efflux [42]. These microorganisms are known as metal excluders [46]. In contrast, higher Cd removal in *Aspergilus versicolor* may relate to accumulation of cadmium in cell structures. Fungal biomass can act as a metal sink, either by: (1) metal biosorption to biomass (cell walls, pigments and extracellular polysaccharides); or (2) intracellular accumulation and sequestration; or (3) precipitation of metal compounds onto and/or around hyphae [42,47].

Fungi were known to accumulate significant amount of cadmium, for example uptake concentrations of 6.46 mggˉ^1^by *Aspergillus* nijer and 16.25 mg gˉ^1^ by *Trichoderma viride* have been reported earlier [48]. *Pisolithus tinctorius* presented maximal uptake of 600 mg kgˉ^1^ dry weight at 10 mg lˉ^1^ cadmium concentration [5].

## Conclusion

The present study declared seven highly tolerant fungi . These fungi exhibited various resistance strategies towards cadmium and they had an ability to sequester cadmium from liquid media.

*Aspergilus versicolor* remarkably differed in detoxification behavior from other isolated fungi in this study. The fungus showed a remarkable potential to actively grow in presence of Cd and reduce cadmium concentration to less toxic levels. Introducing *Aspergilus versicolor* as scavenger biota is the first step of emerging this fungus in bioremediation science.

Efforts are being made to make bioremediation technically/economically feasible; therefore, we should direct our attention to exploit whole potential of microorganism.

Understanding metal uptake process genetically, manipulation of cell structure such as autoclaving or drying biomass, and using combo strains are innovative technologies in biotreatment studies.
